# The characteristics and health needs of pregnant women with schizophrenia compared with bipolar disorder and affective psychoses

**DOI:** 10.1186/s12888-015-0451-8

**Published:** 2015-04-17

**Authors:** Clare L Taylor, Robert Stewart, Jack Ogden, Matthew Broadbent, Dharmintra Pasupathy, Louise M Howard

**Affiliations:** Section of Women’s Mental Health/Women’s Health Academic Centre, Department of Health Service and Population Research, King’s College London, Institute of Psychiatry, London, UK; Department of Psychological Medicine, King’s College London, Institute of Psychiatry, London, UK; South London and Maudsley NHS Foundation Trust, London, UK; Women’s Health Academic Centre, King’s College London, London, UK

**Keywords:** Schizophrenia, Bipolar disorder, Pregnancy

## Abstract

**Background:**

Most women with psychotic disorders and bipolar disorders have children but their pregnancies are at risk of adverse psychiatric and fetal outcome. The extent of modifiable risk factors – both clinical and socio-demographic – is unclear as most studies have used administrative data or recruited from specialist tertiary referral clinics. We therefore aimed to investigate the socio-demographic and clinical characteristics of an epidemiologically representative cohort of pregnant women with affective and non-affective severe mental illness.

**Methods:**

Women with severe mental illness were identified from a large electronic mental health case register in south London, and a data linkage with national maternity Hospital Episode Statistics identified pregnancies in 2007–2011. Data were extracted using structured fields, text searching and natural language processing applications.

**Results:**

Of 456 pregnant women identified, 236 (51.7%) had schizophrenia and related disorders, 220 (48.3%) had affective psychosis or bipolar disorder. Women with schizophrenia and related disorders were younger, less likely to have a partner in pregnancy, more likely to be black, to smoke or misuse substances and had significantly more time in the two years before pregnancy in acute care (inpatient or intensive home treatment) compared with women with affective disorders. Both groups had high levels of domestic abuse in pregnancy (recorded in 18.9%), were from relatively deprived backgrounds and had impaired functioning measured by the Health of the Nation Outcome Scale. Women in the affective group were more likely to stop medication in the first trimester (39% versus 25%) whereas women with non-affective psychoses were more likely to switch medication.

**Conclusions:**

A significant proportion of women, particularly those with non-affective psychoses, have modifiable risk factors requiring tailored care to optimize pregnancy outcomes. Mental health professionals need to be mindful of the possibility of pregnancy in women of childbearing age and prescribe and address modifiable risk factors accordingly.

**Electronic supplementary material:**

The online version of this article (doi:10.1186/s12888-015-0451-8) contains supplementary material, which is available to authorized users.

## Background

Most women with severe mental illnesses (SMI; i.e. schizophrenia and related delusional disorders, and bipolar disorder and affective psychoses) have children [1], and with the use of newer antipsychotic medications that do not raise prolactin, fertility in women with SMI is increasing [2,3]. However SMI, whether affective or non-affective, is associated with a range of adverse consequences in pregnancy and the postpartum period.

Firstly acute psychiatric relapse may occur - internationally, the Confidential Enquiries into Maternal Deaths highlight psychiatric illness as a leading contributor to maternal deaths [4,5] - and postpartum relapse may also affect capacity to parent the infant [6]. Pregnancy is associated with discontinuation of psychotropic medication in women with severe affective disorders [7-9] but there is little known about rates and reasons for discontinuation in women with schizophrenia.

There is also increasing evidence that adverse fetal outcomes, including small for gestational age babies, prematurity and stillbirth, are associated with SMI [10-13], and that these outcomes are at least partly due to risk factors associated with SMI including socio-demographic factors [1], smoking and substance misuse [10], nutritional deficiencies [14], obesity [15] and domestic violence [16]. However the prevalence of these modifiable risk factors in these women is unclear as most studies have used clinical data from specialist services with limited generalizability, or have small clinical samples with limited statistical power to investigate differences between the groups [17], or have used administrative data of population cohorts with little detail on clinical characteristics. It is also not clear to what extent these risk factors differ in pregnant women with schizophrenia and related delusional disorders compared with women with bipolar disorder or affective psychoses.

We therefore aimed to establish a more representative cohort of pregnant women with SMI, using detailed electronic clinical health records. This would enable us to investigate the prevalence of socio-demographic and clinical risk factors, and establish whether these differ in women with affective and non-affective SMI, in order to inform service providers of this population’s health needs. Given that schizophrenia is associated with poorer physical and psychosocial functioning than affective psychosis, we hypothesised that, compared with pregnant women with non-affective SMI, pregnant women with affective SMI would have fewer behavioural risk factors associated with adverse pregnancy outcomes (e.g. smoking, domestic violence, substance misuse), have better social support, fewer episodes of acute care prior to pregnancy and be less likely to stop or switch medication during pregnancy.

## Methods

### Data Source

The South London and Maudsley (SLaM) NIHR Biomedical Research Centre Clinical Record Interactive Search (CRIS) system is a ‘new generation’ of case register design, built on fully electronic clinical records (maintained since 2006), preserving anonymity through technical and procedural safeguards [18]. SLaM provides near-monopoly mental healthcare for 1.2 million people, as well as specialist services. CRIS, described in detail elsewhere [19], allows searching and retrieval of anonymised full records from SLaM including copies of text fields (e.g. case notes, correspondence) with masking of identifiers [18]. CRIS currently accesses data on over 250,000 individuals. Data can be extracted from structured fields, or search terms can be entered to perform targeted searches of the notes and correspondence. Several natural language processing applications have been developed using General Architecture for Text Engineering (GATE) software in collaboration with Sheffield University Department of Computer Science. These applications derive structured data from free text fields, taking into account the linguistic context. Applications have been developed and validated against human raters [20]. CRIS was approved as a source of secondary data for research by Oxfordshire Research Ethics Committee C (08/H0606/71 + 5). Information can only be reported where there are more than five participants in a cell to maintain anonymity.

Using a trusted third party in full compliance with UK Data Protection law, CRIS data have been linked with Hospital Episode Statistics (HES) [21], which provide national statistical data on all hospital care in England, including hospital admissions, outpatient appointments, Emergency Room attendances and maternity care.

### Study design and population

A cohort of women who were pregnant whilst receiving SLaM care at any point from 2007–2011 inclusive was assembled using CRIS and HES. Women with SMI were eligible if they have ever had the following ICD-10 [22] diagnoses recorded on the CRIS database: F20, F22, F23, F25, F28, F29 (schizophrenia and related disorders, schizoaffective disorders and delusional disorders), F30, F31 (mania and bipolar affective disorders), F32.3, F33.3 (psychotic depression), F53.1 (severe mental and behavioural disorders associated with the puerperium – specifically puerperal psychosis). The HES linkage was used to identify incidences of pregnancy in these women during the study period. All women with first episode psychosis occurring after or during the index pregnancy were then excluded.

Diagnosis at baseline was assigned by extracting the closest SMI diagnosis to 9 months before the HES episode using CRIS structured fields and GATE software. Where no diagnoses were extracted, notes and correspondence histories were searched. Dating of pregnancy was carried out using gestational age at birth extracted from HES maternity data and used as our ‘gold standard’. Where this was unavailable, manual text searches in CRIS were carried out for expected delivery dates or other indicators using search terms related to pregnancy and birth. For the remaining pregnancies, we adapted a published and validated algorithm for estimating gestational age at birth in electronic health plan databases [8,23] - full term pregnancies were assigned a 270-day gestational length and preterm pregnancies 245 days; first trimester was identified as 0–89 days [23]. For the purposes of the current analysis, we used the first index pregnancy occurring in the study period.

### Measures

#### Socio-demographic characteristics for index (first) pregnancy

We extracted ethnicity from structured fields and categorised codes into ‘Black African/Caribbean/other’, ‘White British/other’, and ‘Mixed, Asian or other’. Patient addresses on CRIS have been linked to small-area-level UK Census data (2007 projections from the 2001 Census) to provide indices of multiple deprivation [24]; the higher the score, the more severe the deprivation (in the 2007 report, indices range from 0.37 to 85.46 [24]). Manual searches were carried out to establish number of other children prior to index pregnancy, partner status, smoking, alcohol and drug use during pregnancy, recorded history of maternal abuse in childhood and whether the woman had been a victim of domestic abuse before and during pregnancy (encompassing physical, sexual, emotional and coercive abuse).

#### Clinical characteristics

Deliberate self-harm in the two years prior to pregnancy was ascertained from manual review of notes and correspondence. Number of days in acute mental health care in the two years before pregnancy (defined as an inpatient episode or under the care of intensive home treatment teams i.e. care provided at home by teams that are available everyday who can visit up to three times per day [25]) were extracted from structured fields (or manual review of clinical text fields for pre-2006 period), supplemented by HES data for other (non-SLaM) mental health services. Spells of acute care were constructed whereby a spell comprised of a period of treatment in acute mental health care (inpatient or home treatment) where there was at least 7 days between admissions and discharges. Time since last major episode was calculated as the time from the beginning of the most recent ‘spell’ of acute care in the 2 years prior to conception.

Baseline level of functioning recorded in the two years before pregnancy was estimated by using the highest total adjusted score from the Health of the Nation Outcome Scale (HoNOS), a routinely collected 12 item measure in UK mental health services of health and social functioning of people with severe mental illness. Patients with SMI typically have a score of around 10 indicating clinically significant limitation in functioning; patients under acute care typically have scores around 14 [26].

The GATE medication application was used to extract structured indicators describing medication from the free text for three months before pregnancy and the first trimester of pregnancy. This was used to guide the retrieval of relevant clinical text fields containing medication information for in-depth manual examination. Stops, starts and switches in regular medication(namely antipsychotics, mood stabilisers and antidepressants) were noted and the recorded reasons for these. Two researchers carried out these manual reviews on randomly split portions of the sample with consensus meetings to resolve uncertainties. Two raters (CT, JO) cross-checked 5 cases each week until satisfactory reliability was obtained and then a consecutive 22 cases (26 pregnancies) were independently rated for reliability analyses

### Statistical analysis

Analyses were carried out using STATA, version 12. Women were stratified into two main diagnostic groups – non-affective SMI, comprising those with baseline diagnoses of schizophrenia, delusional disorders, acute and transient psychoses, schizoaffective disorders, other non-organic psychoses and psychosis NOS - and affective SMI, including bipolar affective disorder & manic episodes, psychotic depression and history of postpartum psychosis. As the classification of schizoaffective disorders varies depending on the classification system used (DSM-IIIR,-IV and -V criteria for schizoaffective disorder are closer to schizophrenia, whereas ICD-10 criteria seem to allow the inclusion of a broader, more heterogeneous group of patients into the diagnosis [27]), and is still the subject of debate, analysis was repeated by including women with schizoaffective disorder in the affective group as a sensitivity analysis.

Descriptive analyses investigated demographic characteristics and psychiatric history for the whole sample and separately for each of the two diagnostic groupings, using chi^2^ tests for categorical data, t-tests for normally distributed data and non-parametric Mann–Whitney tests for other continuous data. For this analysis, cases with missing smoking, alcohol, or substance use status (defined as any use in pregnancy) and non-users were combined so we compared only those who were identified as users in pregnancy. Prevalence of medication use was recorded for each trimester for medication groups and each individual medication. Comparisons were made using chi^2^ tests and chi^2^ tests for trend were used to examine trends in medication by year of pregnancy. Where women had more than one pregnancy over the duration of the study the first (index) pregnancy was used for this analysis. Inter-rater reliabilities were assessed by calculating percent agreement for identification of antipsychotics, antidepressants and mood stabilisers.

## Results

### Socio-demographic and clinical characteristics

**Figure 1:**
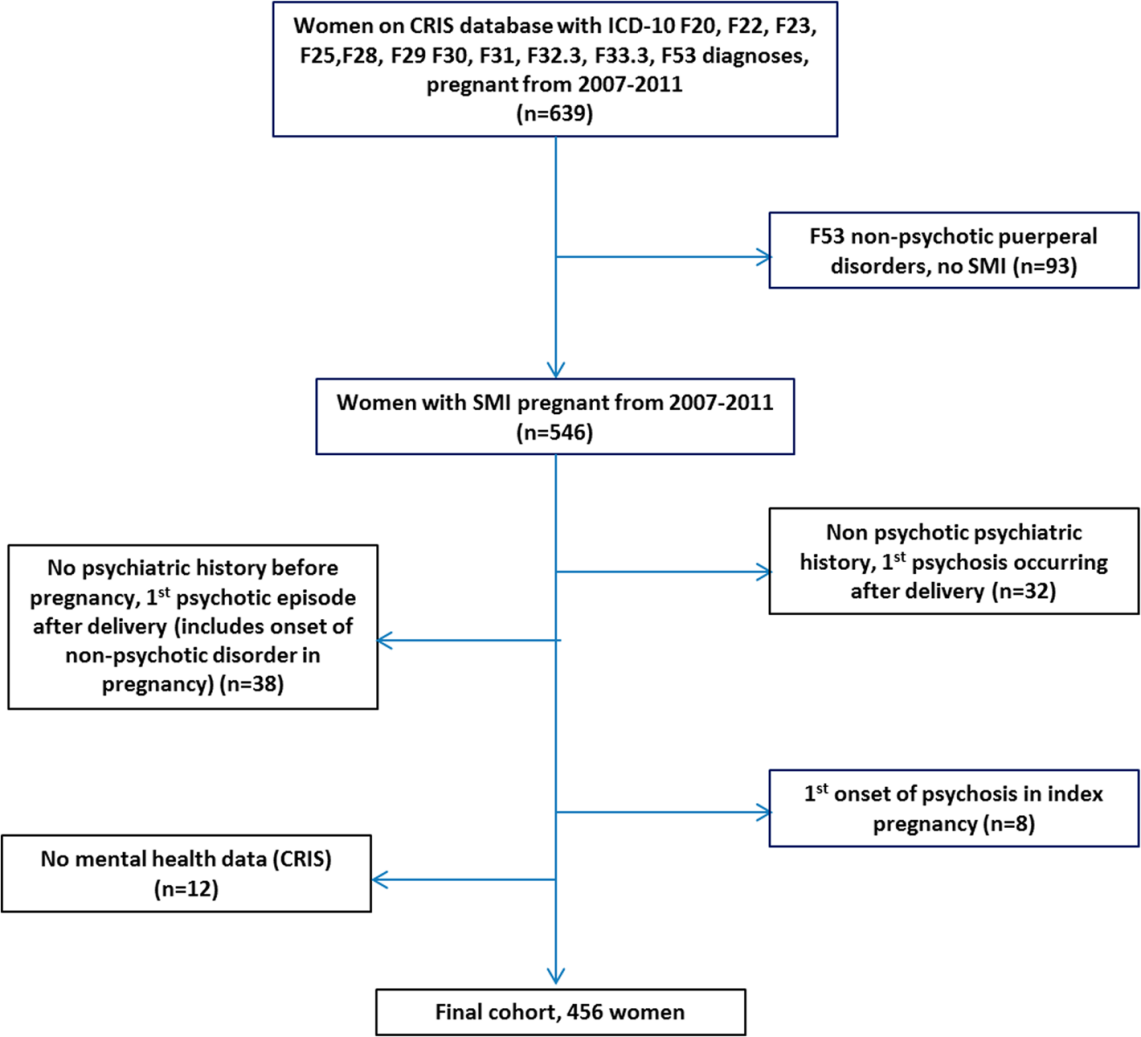
Identification of women with serious mental illness.

We identified 456 women with SMI with 539 pregnancies during the study period 2007–2011 (Figure 1); 68 women had more than one pregnancy in the study period. Using diagnoses recorded closest to the beginning of the first (index) pregnancy, there were 236 (51.7%) women with schizophrenia and related disorders (including 31 with schizoaffective diagnoses), and 220 (48.3%) women with affective SMI (165 bipolar affective disorder, 48 psychotic depression, and 7 with a history of postpartum psychosis only). Secondary diagnoses recorded included 24 women (5.3%) with substance use disorders, 13 (2.9%) with anxiety disorders including obsessive compulsive disorder and post-traumatic stress disorder, 13 (2.9%) with personality disorders, 5 (1.1%) with pervasive learning difficulties, and 6 (1.3%) with “other” diagnoses including depressive disorders, epilepsy, eating disorders and conduct disorders.

There was no difference in the duration of CRIS records available between the two diagnostic groups: median (range) durations were 817 (0–5006) and 834.5 (0–5500) days for non-affective and affective SMI respectively (p = 0.419).

Women with non-affective SMI were significantly more likely to be of Black African or other black ethnicity, younger, current smokers and using illicit substances during pregnancy, and had more acute admissions and more time in acute care in the two years before pregnancy compared with the affective group (Table 1).

**Table 1:** **Clinical and demographic characteristics of pregnant women with severe mental illness**

	**Whole sample N = 456**	**Non-affective psychosis, N = 236**	**Affective SMI, N = 220**	P*
**Ethnicity**				
Black African & other Black	221 (48.5)	137 (58.1)	84 (38.2)	**<0.001**
White British & other White	152 (33.3)	58 (24.6)	94 (42.7)	
Asian/ Mixed/Other	83 (18.2)	41 (17.4)	42 (19.1)	
**Deprivation score, median (range)** ^**1**^	34.9 (3.8-77.2)	35.4 (3.8-77.2)	33.6 (6.8-9.7)	0.226
**Maternal age at 1** ^**st**^ **index delivery, mean (SD),**	31.8 (6.2)	30.9 (6.4)	32.9 (5.8)	**<0.001**
**Partner during 1** ^**st**^ **index pregnancy**				
Yes	299 (68.7)	143 (63.8)	156 (73.9)	**0.023**
No	136 (31.3)	81 (36.2)	55 (26.1)	
**Number of children at 1** ^**st**^ **index pregnancy**				
0	197 (44.7)	101 (43.0)	96 (46.6)	0.682
1	125 (28.3)	71 (30.2)	54 (26.2)	
2	68 (15.4)	38 (16.2)	30 (14.6)	
>/=3	51 (11.6)	25 (10.6)	26 (12.6)	
**Victim of child abuse**	106 (23.3)	60 (25.4)	46 (20.9)	0.254
**Victim of domestic abuse before pregnancy**	159 (34.9)	83 (35.2)	76 (34.6)	0.889
**Victim of domestic abuse in pregnancy**	86 (18.9)	45 (19.1)	41 (18.6)	0.906
**Smoking in pregnancy**	79 (17.3)	51 (21.6)	28 (12.7)	**0.012**
**Alcohol use in pregnancy**	77 (16.9)	40 (17.0)	37 (16.8)	0.970
**Substance use in pregnancy**	61 (13.4)	39 (16.5)	22 (10.0)	**0.041**
**Self-harm in 2 years before pregnancy**	67 (14.7)	41 (17.4)	26 (11.8)	0.094
**Number of days of acute care in 2 years before pregnancy,**				
0	262 (57.5)	121 (51.3)	141 (64.1)	**0.021**
1-33	67 (14.7)	38 (16.1)	29 (13.2)	
34-79	63 (13.8)	42 (17.8)	21 (9.6)	
83-537	64 (14.0)	35 (14.8)	29 (13.2)	
**Number of acute admissions in 2 years before pregnancy**				
0	262 (57.5)	121 (51.3)	141 (64.1)	**0.025**
1	127 (27.9)	78 (33.1)	49 (22.3)	
2	38 (8.3)	23 (9.8)	15 (6.8)	
>2	29 (6.4)	14 (5.9)	15 (6.8)	
**Time since last admission (years)**				
1 year	104 (53.6)	65 (56.5)	39 (49.4)	0.326
2 years	90 (46.4)	50 (43.5)	40 (50.6)	
**Highest HoNOS total adjusted score in 2 years before pregnancy, median (range)** ^**2**^	12 (0–36)	12 (0–36)	12 (0–28)	0.768

^1^Deprivation score, whole sample, n = 427, non-affective group, n = 222, affective group, n = 205.

^2^adjusted HoNOS, whole sample, n = 244, non-affective group, n = 146, affective group, n = 98.

*p values are for comparison between the affective and non-affective group.

### Medication

Agreement between the two raters was 92% - 100%. Six pregnancies had missing medication data in the first trimester. Table 2 summarises medication exposures in the three months prior to pregnancy, and in the first trimester of pregnancy. The most common antipsychotic exposure was olanzapine. Rates of polypharmacy were similar in affective (44.8%) compared to non-affective SMI (38.9%) (chi^2^(1) = 1.11, p = 0.291). Of the regular medication prescribed (antipsychotics, mood stabilisers and antidepressants), antipsychotics were the most common medication exposure and more common in the non-affective than affective SMI group, both in the 3 months before pregnancy (chi^2^(1) = 27.22, p < 0.001) and in the first trimester of pregnancy (chi^2^(1) = 31.15, p < 0.001).

**Table 2:** **Recorded psychotropic medication use before/during early pregnancy in women with severe mental illness**

**Medication**	**90 days pre pregnancy, N (%)**			**1** ^**st**^ **trimester, N (%)**		
	**Whole sample**	**Non-affective**	**Affective**	**Whole sample**	**Non-affective**	**Affective**
	**N = 450**	**N = 234**	**N = 216**	**N = 445**	**N = 232**	**N = 213**
**No regular medication**	126 (28.0)	61 (26.1)	65 (30.1)	133 (29.9)	65 (28.0)	68 (31.9)
**Antipsychotics**	255 (56.7)	160 (68.4)	95 (44.0)	247 (55.5)	158 (68.1)	89 (41.8)
**Olanzapine**	98 (21.8)	52 (22.2)	46 (21.3)	97 (21.8)	53 (22.8)	44 (20.7)
**Risperidone**	56 (12.4)	37 (15.8)	19 (8.8)	52 (11.7)	36 (15.2)	16 (7.5)
**Aripiprazole**	45 (10.0)	40 (17.1)	5 (2.3)	39 (8.8)	34 (14.7)	5 (2.4)
**Quetiapine**	35 (7.8)	14 (6.0)	21 (9.7)	29 (6.5)	11 (4.7)	18 (8.5)
**Haloperidol**	16 (3.6)	8 (3.4)	8 (3.7)	23 (5.2)	17 (7.3)	6 (2.8)
**Trifluoperazine**	8 (1.8)	6 (2.6)	<5	9 (2.0)	7 (3.0)	<5
**Chlorpromazine**	<5	<5	<5	7 (1.6)	<5	<5
**Flupenthixol**	8 (1.8)	8 (3.4)	0	8 (1.8)	8 (3.5)	0
**Other** ^**1**^	24 (5.3)	18 (7.7)	6 (2.8)	23 (5.2)	18 (7.8)	5 (2.4)
**Mood stabilisers**	82 (18.2)	21 (9.0)	61 (28.2)	74 (16.6)	18 (7.8)	56 (26.3)
**Sodium Valproate**	41 (9.1)	15 (6.4)	26 (12.0)	34 (7.6)	13 (5.6)	21 (9.9)
**Lithium**	24 (5.3)	<5	22 (10.2)	21 (4.7)	<5	20 (9.4)
**Lamotrigine**	11 (2.4)	<5	8 (3.7)	12 (2.7)	<5	9 (4.2)
**Carbamazepine/Topimarate**	9 (2.0)	<5	6 (2.8)	9 (2.0)	<5	6 (2.8)
**Antidepressants**	107 (23.8)	39 (16.7)	68 (31.5)	103 (23.2)	37 (16.0)	66 (31.0)
**Citalopram/ Escitalopram**	25 (5.6)	11 (4.7)	14 (6.5)	28 (6.3)	11 (4.7)	17 (8.0)
**Fluoxetine**	28 (6.2)	7 (3.0)	21 (9.7)	25 (5.6)	8 (3.5)	17 (8.0)
**Sertraline**	15 (3.3)	8 (3.4)	7 (3.2)	17 (3.8)	9 (3.9)	8 (3.8)
**Venlafaxine**	18 (4.0)	5 (2.1)	13 (6.0)	16 (3.6)	5 (2.2)	11 (5.2)
**Mirtazepine**	15 (3.3)	<5	11 (5.1)	11 (2.5)	<5	7 (3.3)
**Other** ^**2**^	16 (3.6)	7 (3.0)	9 (4.2)	17 (3.8)	5 (2.2)	12 (5.6)
Polypharmacy*						
**2 agents**	104 (23.1)	49 (20.9)	55 (25.5)	97 (21.8)	49 (21.1)	48 (22.5)
**>2 agents**	31 (6.9)	13 (5.6)	18 (8.3)	33 (7.4)	13 (5.6)	17 (8.0)
**Anxiolytics** ^**3**^	40 (7.5)			34 (6.5)		
**Lorazepam**	13 (2.9)	9 (3.9)	4 (1.9)	9 (2.0)	5 (2.2)	<5
**Diazepam**	10 (2.2)	<5	8 (3.7)	8 (1.8)	<5	5 (2.4)
**Other** ^**3**^	14 (3.1)	7 (3.0)	7 (3.2)	12 (2.7)	<5	8 (3.8)
**Sedatives**	35 (7.8)	15 (6.4)	20 (9.3)	37 (8.3)	16 (6.9)	21 (9.9)
**Promethazine**	15 (3.3)	8 (3.4)	7 (3.2)	21 (4.7)	10 (4.3)	11 (5.2)
**Zopiclone/zolpidem**	23 (5.1)	9 (3.9)	14 (6.5)	24 (5.4)	9 (3.9)	15 (7.0)
**Side effects medication**						
**Procyclidine**	18 (4.0)	10 (4.3)	8 (3.7)	14 (3.2)	9 (3.9)	5 (2.4)

^1^antipsychotic “other” includes: , clozapine (n = 5), amisulpride, clopixol, piportil, prochlorperazine, sulpride.

^2^antidepressant “other” includes: amitriptiline, dosulepin, dothiepin, duloxetine, imipramine, maprotiline, paroxetine, repoxetine, trancyclopromine, trazodone, trimipramine, tryptophan, lofepramine, selegiline.

^3^anxiolytics “other” includes: clonazepam, midazolam, chlordiazepoxide.

*refers to any combination of antidepressants, mood stabilisers and antipsychotic.

Fifteen percent of pregnancies were exposed to mood stabilisers, with 50% discontinuation rates in the first trimester. Mood stabilisers were more commonly prescribed for affective SMI in the 3 months before pregnancy (chi^2^(1) = 27.98, p < 0.001) and during the first trimester (chi^2^(1) = 27.51, p < 0.001). The most commonly prescribed mood stabiliser was sodium valproate (7.6% of all index pregnancies in the first trimester). Antidepressants were more commonly prescribed in the affective than non-affective group in the 3 months before pregnancy (chi^2^(1) = 13.60, p < 0.001) and during the first trimester (chi^2^(1) = 14.12, p < 0.001). The most common antidepressants prescribed were citalopram/escitalopram, fluoxetine and sertraline, followed by venlafaxine and mirtazapine.

A high proportion (40.4%) of women stopped or switched medication in the first trimester of pregnancy, particularly mood stabilisers (Table 3). Women with affective SMI were more likely to stop medication in the first trimester compared with women with non-affective SMI (chi^2^(1) = 6.54, p = 0.011), while women with non-affective SMI were more likely to switch medication in the first trimester of pregnancy (chi^2^(1) = 5.47, p = 0.019). There was no difference between the affective and non-affective groups in the proportion of women who *either* stopped *or* switched medication in the first trimester of pregnancy (chi^2^(1) = 0.63, p = 0.426). In terms of the reasons why the 98 women stopped medication in the first trimester, 78.6% were recorded as stopping because of the “pregnancy”, 7.1% due to “side effects of medication”, 5.1% were recorded as “non-compliance”, and 5.1% as due to symptoms improving. Eighty nine (90.8%) stopped abruptly and 9 (9.2%) stopped gradually; where patients had decided themselves to stop medication they all stopped medication abruptly (n = 56), whereas for those that stopped after discussion with a clinician, 30(76.9%) stopped abruptly. Thirty three women switched medication in the first trimester with the commonest reasons being because of “pregnancy” (63.6%) and “side effects” (12.2%).

**Table 3:** **Regular medication changes in first trimester (n (%))**

	**Whole sample**	**Non-affective psychosis**	**Affective SMI**
Antipsychotics	*N = 247*	*N = 158*	*N = 89*
Switched	28 (11.3)	21 (13.3)	7 (7.9)
Stopped	59 (24.0)	33 (21.9)	26 (29.2)
Stopped or switched	84 (34.0)	53 (33.5)	31 (34.8)
Mood stabilisers	*N = 74*	*N = 18*	*N = 56*
Switched	<5	0	<5
Stopped	36 (48.7)	9 (50.0)	27 (48.2)
Stopped or switched	37 (50.0)	9 (50.0)	28 (50.0)
Antidepressants	*N = 103*	*N = 37*	*N = 66*
Switched	<5	<5	<5
Stopped	27 (26.2)	7 (18.9)	20 (30.3)
Stopped or switched	30 (29.1)	9 (24.3)	21 (31.8)
Regular medication	*N = 312*	*N = 167*	*N = 145*
Switched	32 (10.3)	23 (13.9)	9 (6.2)
Stopped	98 (31.4)	42 (25.2)	56 (38.6)
Stopped or switched	125 (40.1)	63 (37.7)	62 (42.8)

Disaggregated by year of pregnancy, for the proportion of women being prescribed antipsychotic medication over time - 47.7% of 1^st^ index pregnancies in 2007 had first trimester exposure to antipsychotics, 52.8% of pregnancies in 2008, 58.7% of pregnancies in 2009, 59.2% in 2010 and 60.0% in 2011 (Chi^2^(1) =3.35, p = 0.067). Regarding mood stabilisers in the first trimester, 20.9% were exposed in 2007, 20.8% in 2008, 14.1% in 2009, 11.3% in 2010 and 14.4% in 2011 (Chi^2^(1) = 3.12, p = 0.078. For antidepressant medication use during the study period - in 2007, 22.1% were exposed, 20.8% in 2008, 26.1% in 2009, 25.4% in 2010, and 22.2% in 2011 (Chi^2^(1) =0.12, p = 0.734).

A sensitivity analysis where schizoaffective disorder was classified with affective SMI did not result in meaningful changes other than for self-harm which was significantly more common in the affective SMI group categorised in this way, and for the number of days in acute care and number of admissions before pregnancy where differences were no longer statistically significant (Additional file 1: Table S4).

## Discussion

### Main findings

We identified a cohort of 456 women with SMI pregnant from 2007–2011. In this cohort , women with non-affective SMI had significantly higher levels of recent acute psychiatric morbidity, less social support (more likely to be single), were younger and had a higher prevalence of smoking and substance misuse during pregnancy compared with women with affective SMI. This suggests that women with non-affective SMI will need particularly high levels of support to optimise both psychiatric and obstetric adverse outcomes. Pregnant women in both diagnostic groups were from relatively deprived backgrounds, had significantly impaired levels of functioning and had relatively high levels of domestic violence in pregnancy recorded in the clinical record. Statistics from the local area show smoking in pregnancy to be at about 4.4% in the local catchment area [28] suggesting our cohort of women with SMI were more likely to smoke in pregnancy. However the prevalence in our sample of women with SMI was much lower than the 38% prevalence reported in a similar clinical population in a retrospective case note review [17]; this is likely to reflect under-reporting by patients and under-recording by clinicians. Some of these risk factors, notably smoking, substance misuse and domestic violence, are potentially modifiable but, although there is evidence for the effectiveness of interventions such as smoking cessation programmes and domestic violence advocacy [29], these are not currently tailored for women with SMI and more evidence is needed on how to best support women with SMI to reduce these risks. The data we have provided on trauma (child abuse and domestic violence) are of particular significance as they could impact on child protection outcomes and the need for trauma informed obstetric care.

Following the onset of pregnancy, a relatively high proportion of women, around 40% in both diagnostic groups, either stopped or switched medication in the first trimester of pregnancy, particularly mood stabilisers. Women in the affective group were more likely than women in the non-affective group to stop medication in the first trimester, but less likely to switch medication. Similar levels of changes in antipsychotic use have been described in data from USA health plans [8] and women in contact with a German teratology service [30]. Around fifteen percent of pregnancies in this study involved exposure to mood stabilisers, mainly sodium valproate and lithium, with 50% discontinuation rates in the first trimester; similar discontinuation rates have been described in the USA [8] and in UK primary care [31]. Our data on the extent of medication exposure in early pregnancy therefore appears to be consistent with other international studies and reflects concerns by both clinicians and patients of teratogenicity. As teratogenic effects are most likely in the first few weeks of pregnancy, stopping mood stabilisers when women know they are pregnant may be too late to prevent these effects, although the impact on neurodevelopment continues for valproate exposure throughout pregnancy [32], so discontinuation would still be indicated. Nevertheless, as pregnancy is unplanned in 50-70% of pregnancies in women with SMI it may be safer not to prescribe valproate to women of childbearing age other than when other medication options have failed – and NICE and SIGN guidelines do indeed recommend this avoidance [33,34]. NICE 2007 guidelines also recommend considering an antipsychotic as an alternative. This appears to be reflected in clinical practice here as there were increases in antipsychotic exposures and decreases in mood stabilisers in the changes in exposure to medication during pregnancy over time from 2007–2011.

The impact of switching and discontinuation of medication is not clear as the limited literature to date has been based on selected clinical samples of pregnant women with bipolar disorder in whom discontinuation of mood stabilisers was associated with a marked increased risk of relapse during pregnancy after adjusting for potential confounders [9]. Outside of the perinatal period switching antipsychotics in people with bipolar disorder [35] or schizophrenia [36,37] appears to be well tolerated but discontinuation is associated with increased risk of relapse, particularly where discontinuation is carried out abruptly [9,38]. Women may be unaware that most psychotropic medication prescribed is associated with very small absolute risks in pregnancy while discontinuation, particularly abrupt discontinuation, may lead to relapse. Follow up of this cohort of women will enable us to investigate the extent to which discontinuation or switching of medication is associated with relapse in both women with affective or non-affective psychoses but this data suggests that psycho-education on the risks of abrupt discontinuation in pregnancy is needed. We will also be able to investigate obstetric outcomes.

Given that most women with SMI have children, and fertility rates have been shown to be increasing [1-3], our findings imply that more attention should be paid to reproductive health when prescribing medications for SMI in women of child bearing age. A high proportion of women changed medication once pregnant. It is noteworthy that in women taking the same medications for epilepsy, far lower rates of discontinuation occur [31], suggesting different attitudes to risk and benefit, despite the fact that maternal deaths are more commonly associated with mental disorders than epilepsy [4]. It is therefore important for psychiatric services to implement recommendations for pre-conception counselling for women with pre-existing chronic conditions so that women can make better informed decisions about medication and support with other risk factors before conception to optimise pregnancy outcomes.

### Strengths and limitations

A major strength of this data is the size and likely generalisability of the sample. The mental health provider (South London and Maudsley NHS Foundation Trust) provides near monopoly coverage of its geographical area enabling us to have established a cohort of representative women from an ethnically diverse population with SMI in the perinatal period. However it includes only women in receipt of secondary care, thus missing cases managed exclusively in primary care. Although these data come from the medical notes of patients in one secondary mental health care provider and may thus simply reflect the patterns of prescribing of clinicians in a particular health care trust, the similar rates of medication discontinuation in pregnancy to other national and international data suggests these data are more generalizable to other areas and services.

In addition the CRIS database reflects a dynamic cohort of women who may be referred in and out of services and move from the catchment area; the use of Hospital Episode Statistics, with full coverage of England, allowed follow up for women who moved away or were discharged and enabled us to collect data for women who moved into the area.

Multiple statistical comparisons were made suggesting some associations could have occurred by chance. However findings were in the expected direction suggesting face validity and increasing evidence that our women are a representative cohort of pregnant women with SMI.

The linkage to maternity data also provided a robust method of identifying pregnancies including the whole of England; however, maternity information has varying levels of completeness, some systematic errors in its transfer [39] and it does not include births taking place at home, which was 2.4% for England in 2011 [40]. Finally by using the full clinical care records, a high level of clinical detail can be collected. In particular we collected more detailed data regarding medication use in pregnancy than in many previous studies which have used prescription registries and health plan data only. However the quality of clinical data depends on the accuracy and comprehensiveness of clinicians’ records and some important data are missing such as Body Mass Index and detailed descriptions of social support.

## Conclusions

A range of potentially modifiable risk factors for adverse outcomes of pregnancy and childbirth were identified in this cohort of women with SMI, with women with non-affective psychoses at particularly high risk. Health providers should develop strategies for addressing these risk factors, for example by discussing smoking cessation and referring to domestic violence advocacy before and during pregnancy. However, research is still needed to establish whether and how these interventions need to be modified for this population. Optimisation of medication similarly should ideally take place before conception; prescribers of medication to women of childbearing age need to be mindful of the possibility of pregnancy and prescribe accordingly.

## Additional file

Additional file 1: Table S4. Clinical and demographic characteristics of cohort of pregnant women with severe mental illness (with schizoaffective disorder in affective group).

## Abbreviations

SMI: Severe mental illness
SLaM: South London and Maudsley NHS Foundation Trust
CRIS: Clinical Record Interactive Search
GATE: General Architecture for Text Engineering
HES: Hospital Episode Statistics
ICD: International Classification of Diseases
DSM: Diagnostic and Statistical Manual of Mental Disorders
HoNOS: Health of the Nation Outcome Scales
UK: United Kingdom
NOS: Not otherwise specified
NICE: UK National Institute of Health and Care Excellence
SIGN: Scottish Intercollegiate Guidelines Network
NHS: UK National Health Service

## References

[1] Howard LM, Kumar R, Thornicroft G (2001). Psychosocial characteristics and needs of mothers with psychotic disorders. Br J Psychiatry.

[2] Howard LM, Kumar C, Leese M, Thornicroft G (2002). The general fertility rate in women with psychotic disorders. Am J Psy.

[3] Vigod SN, Seeman MV, Ray JG, Anderson GM, Dennis CL, Grigoriadis S (2012). Temporal trends in general and age-specific fertility rates among women with schizophrenia (1996–2009): a population-based study in Ontario, Canada. Schizophr Res.

[4] Cantwell R, Clutton-Brock T, Cooper G, Dawson A, Drife J, Garrod D (2011). Saving Mothers’ Lives: reviewing maternal deaths to make motherhood safer: 2006–2008, the eighth report of the confidential enquiries into maternal deaths in the United Kingdom. BJOG.

[5] Austin M, Kildea S, Sullivan E (2007). Maternal mortality and psychiatric morbidity in the perinatal period: challenges and opportunities for prevention in the Australian setting. Med J Aus.

[6] Howard LM (2005). Fertility and pregnancy in women with psychotic disorders. Eur J Obstet Gynecol Reprod Biol.

[7] Petersen I, Gilbert RE, Evans SJ, Man SL, Nazareth I (2011). Pregnancy as a major determinant for discontinuation of antidepressants: an analysis of data from The Health Improvement Network. J Clin Psychiatry.

[8] Toh S, Li Q, Cheetham TC, Cooper WO, Davis RL, Dublin S, et al. Prevalence and trends in the use of antipsychotic medications during pregnancy in the US, 2001–2007: a population-based study of 585,615 deliveries. Arch Womens Ment Health. 2013: p. 1–9. 10.1007/s00737-013-0330-6 PMC3715880 23389622

[9] Viguera AC, Whitfield T, Baldessarini RJ, Newport DJ, Stowe Z, Reminick A (2007). Risk of recurrence in women with bipolar disorder during pregnancy: prospective study of mood stabilizer discontinuation. Am J Psychiatry.

[10] Bodén R, Lundgren M, Brandt L, Reutfors J, Andersen M, Kieler H. Risks of adverse pregnancy and birth outcomes in women treated or not treated with mood stabilisers for bipolar disorder: Population based cohort study. BMJ. 2012. 345. 10.1136/bmj.e7085 PMC3493986 23137820

[11] Maccabe JH, Martinsson L, Lichtenstein P, Nilsson E, Cnattingius S, Murray RM (2007). Adverse pregnancy outcomes in mothers with affective psychosis. Bipolar Disorders.

[12] Webb R, Abel K, Pickles A, Appleby L (2005). Mortality in offspring of parents with psychotic disorders: a critical review and meta-analysis. Am J Psy.

[13] Webb RT, Pickles AR, King-Hele SA, Appleby L, Mortensen PB, Abel KM (2008). Parental mental illness and fatal birth defects in a national birth cohort. Psychol Med.

[14] McColl H, Dhillon M, Howard LM (2013). A systematic review of the nutritional status of women of a childbearing age with severe mental illness. Arch Womens Ment Health.

[15] Molyneaux E, Poston L, Ashurst-Williams S, Howard LM (2014). Obesity and mental disorders during pregnancy and postpartum: a systematic review and meta-analysis. Obstetrics Gynecology.

[16] Howard LM, Oram S, Galley H, Trevillion K, Feder G (2013). Domestic violence and perinatal mental disorders: a systematic review and meta-analysis. PLoS Med.

[17] Judd F, Komiti A, Sheehan P, Newman L, Castle D, Everall I (2014). Adverse obstetric and neonatal outcomes in women with severe mental illness: To what extent can they be prevented?. Schizophr Res.

[18] Fernandes AC, Cloete D, Broadbent MT, Hayes RD, Chang CK, Jackson RG (2013). Development and evaluation of a de-identification procedure for a case register sourced from mental health electronic records. BMC Med Inform Decis Mak.

[19] Stewart R, Soremekun M, Perera G, Broadbent M, Callard F, Denis M (2009). The South London and Maudsley NHS foundation trust biomedical research centre (SLAM BRC) case register: development and descriptive data. BMC Psychiatry.

[20] Sultana J, Chang C, Hayes R, Broadbent M, Stewart R, Corbett A, et al. Associations between risk of mortality and atypical antipsychotic use in vascular dementia: a clinical cohort study. Int J Geriatr Psychiatry, 2014. 10.1002/gps.4101 24633896

[21] Hospital Episode Statistics [http://www.hscic.gov.uk/hes]

[22] Organization WH. The ICD-10 classification of mental and behavioural disorders: clinical descriptions and diagnostic guidelines. 1992.

[23] Li Q, Andrade SE, Cooper WO, Davis RL, Dublin S, Hammad TA (2013). Validation of an algorithm to estimate gestational age in electronic health plan databases. Pharmacoepidemiol Drug Saf.

[24] Noble M, Mclennan D, Wilkinson K, Whitworth A, Exley S, Barnes H (2007). The English indices of deprivation.

[25] Johnson S, Needle J, Bindman JP, Thornicroft G (2008). Crisis resolution and home treatment in mental health.

[26] Wing J, Beevor A, Curtis R, Park S, Hadden S, Burns A (1998). Health of the Nation Outcome Scales (HoNOS), Research and development. Br J Psychiatry.

[27] Pagel T, Franklin J, Baethge C (2014). Schizoaffective disorder diagnosed according to different diagnostic criteria–systematic literature search and meta-analysis of key clinical characteristics and heterogeneity. J Affect Disord.

[28] Smoking in Pregnancy- Southwark data. Health Profiles 2013. http://www.apho.org.uk/resource/view.aspx?RID=126453

[29] Jahanfar S, Janssen Patricia A, Howard Louise M, Dowswell T. Interventions for preventing or reducing domestic violence against pregnant women. In: Cochrane Database Syst Rev. John Wiley & Sons, Ltd; 2013. 10.1002/14651858.CD009414.pub2 23450603

[30] Habermann F, Fritzsche J, Fuhlbrück F, Wacker E, Allignol A, Weber-Schoendorfer C (2013). Atypical antipsychotic drugs and pregnancy outcome: a prospective, cohort study. J Clin Psychopharmacol.

[31] Man SL, Petersen I, Thompson M, Nazareth I (2012). Antiepileptic drugs during pregnancy in primary care: a UK population based study. PLoS One.

[32] Meador KJ, Baker GA, Browning N, Clayton-Smith J, Combs-Cantrell DT, Cohen M (2009). Cognitive function at 3 years of age after fetal exposure to antiepileptic drugs. New Eng J Med.

[33] Scottish Intercollegiate Guidelines Network (SIGN) (2012). Management of perinatal mood disorders. Vol. (SIGN publication no. 127).

[34] National Institute for Health and Clinical Excellence (2007). Antenatal and postnatal mental health: clinical management and service guidance.

[35] Grande I, Bernardo M, Bobes J, Saiz-Ruiz J, Álamo C, Vieta E. Antipsychotic switching in bipolar disorders: a systematic review. Int J Neuropsychopharmacol. 2013:1-11. 10.1017/S1461145713001168 24139622

[36] Rosa F, Schreiner A, Thomas P, Sherif T (2012). Switching patients with stable schizophrenia or schizoaffective disorder from olanzapine to risperidone long-acting injectable. Clin Drug Investig.

[37] Roussidis A, Kalkavoura C, Dimelis D, Theodorou A, Ioannidou I, Mellos E (2013). Reasons and clinical outcomes of antipsychotic treatment switch in outpatients with schizophrenia in real-life clinical settings: the ETOS observational study. Annals General Psy.

[38] Newport DJ, Stowe ZN, Viguera AC, Calamaras MR, Juric S, Knight B (2008). Lamotrigine in bipolar disorder: efficacy during pregnancy. Bipolar Disord.

[39] Murray J, Saxena S, Modi N, Majeed A, Aylin P, Bottle A. Quality of routine hospital birth records and the feasibility of their use for creating birth cohorts. J Public Health 2012. 10.1093/pubmed/fds077 22967908

[40] Births in England and Wales by Characteristics of Birth 2, 2011 http://www.ons.gov.uk/ons/dcp171778_298892.pdf.

