# Supplementary material for: The characteristics and health needs of pregnant women with schizophrenia compared with bipolar disorder and affective psychoses
Source: BMC Psychiatry. 2015 Apr 17;15:88. doi: 10.1186/s12888-015-0451-8 (PMC4406022; doi:10.1186/s12888-015-0451-8)
Supplement: Additional file 1: Table S4. — Clinical and demographic characteristics of cohort of pregnant women with severe mental illness (with schizoaffective disorder in affective group). [file 12888_2015_451_MOESM1_ESM.docx]

**Supplementary Table 4 Clinical and demographic characteristics of cohort of pregnant women with severe mental illness (with schizoaffective disorder in affective group)**

|  | Whole sample  N = 456 | Non-affective psychosis, N=236 | Affective psychosis, N=220 | P* |
| --- | --- | --- | --- | --- |
| Ethnicity |  |  |  |  |
| Black African & other Black Background | 221 (48.5) | 119 (58.1) | 102 (40.6) | **<0.001** |
| White British & other White Background | 152 (33.3) | 51 (24.9) | 101 (40.2) |  |
| Asian/ Mixed/ Other Ethnicity | 83 (18.2) | 35 (17.1) | 48 (19.1) |  |
| Deprivation score, median(range)^1^ | 34.9 (3.8-77.2) | 35.3 (3.8-77.2) | 34.2 (6.8-61.2) | 0.246 |
| Maternal age at 1^st^ index delivery, mean(SD), | 31.8 (6.2) | 30.7 (6.4) | 32.8 (5.9) | **<0.001** |
| Partner during 1^st^ index pregnancy |  |  |  |  |
| Yes | 299 (68.7) | 124 (64.3) | 175 (72.3) | 0.071 |
| No | 136 (31.3) | 69 (35.8) | 67 (27.6) |  |
| Number of children at 1^st^ index pregnancy |  |  |  |  |
| 0 | 197 (44.7) | 89 (43.6) | 108 (45.6) | 0.962 |
| 1 | 125 (28.3) | 60 (29.4) | 65 (27.4) |  |
| 2 | 68 (15.4) | 32 (15.7) | 36 (15.2) |  |
| >2 | 51 (11.6) | 23 (11.3) | 28 (11.8) |  |
| Victim of child abuse | 106 (23.3) | 54 (26.3) | 52 (20.7) | 0.157 |
| Victim of domestic abuse before pregnancy | 159 (34.9) | 74 (36.1) | 85 (33.9) | 0.619 |
| Victim of domestic abuse in pregnancy | 86 (18.9) | 41 (20.0) | 45 (17.9) | 0.574 |
| Smoking in pregnancy | 79 (17.3) | 45 (22.0) | 34 (13.6) | **0.018** |
| Alcohol use in pregnancy | 77 (16.9) | 36 (17.6) | 41 (16.3) | 0.728 |
| Substance use in pregnancy | 61 (13.4) | 35 (17.1) | 26 (10.4) | **0.036** |
| Self-harm in 2 years before pregnancy | 67 (14.7) | 39 (19.0) | 28 (11.2) | 0.018 |
| Number of days of acute care in 2 years before pregnancy, |  |  |  |  |
| 0 | 262 (57.5) | 107 (52.2) | 155 (61.8) | **0.159** |
| 1-33 | 67 (14.7) | 32 (15.6) | 35 (13.9) |  |
| 34-79 | 63 (13.8) | 35 (17.1) | 39 (11.2) |  |
| 83-537 | 64 (14.0) | 31 (15.1) | 33 (13.2) |  |
| Number of acute admissions in 2 years before pregnancy |  |  |  |  |
| 0 | 262 (57.5) | 107 (52.2) | 155 (61.8) | **0.089** |
| 1 | 127 (27.9) | 66 (32.2) | 61 (24.3) |  |
| 2 | 38 (8.3) | 21 (10.2) | 17 (6.8) |  |
| >2 | 29 (6.4) | 11 (5.4) | 18 (7.2) |  |
| Time since last admission (years) |  |  |  |  |
| 1 year | 104 (53.6) | 65 (56.5) | 39 (49.4) | 0.326 |
| 2 years | 90 (46.4) | 50 (43.5) | 40 (50.6) |  |
| Highest HoNOS total adjusted score in 2 years before pregnancy, median (range)^2^ | 12 (0-36) | 12 (0-36) | 12 (0-29) | 0.996 |

^1^Deprivation score, whole sample, n=427, non-affective group, n=193, affective group, n=234

^2^ adjusted HoNOS, whole sample, n=244, non-affective group, n=129, affective group, n=115
